# Alterations of the immune microenvironment with age predicts patient prognosis of gastrointestinal tract tumours

**DOI:** 10.1002/ctm2.70592

**Published:** 2026-01-07

**Authors:** Fangzhen Li, Jingjing Chen, Junjie Wang, Qiuhong Zhu, Cuiying Chu, Zhiwen Zhang, Yuting Deng, Liang Zhang, Xu Lu, Wei Wang, Huipeng Wang, Dongxue Li, Aili Zhang, Hai‐bo Wu, Wenchao Zhou

**Affiliations:** ^1^ Department of Pathology Centre for Leading Medicine and Advanced Technologies of IHM The First Affiliated Hospital of USTC Division of Life Sciences and Medicine University of Science and Technology of China Hefei Anhui China; ^2^ Intelligent Pathology Institute The First Affiliated Hospital of USTC Division of Life Sciences and Medicine University of Science and Technology of China Hefei Anhui China; ^3^ Department of Neurosurgery the First Affiliated Hospital of USTC Division of Life Sciences and Medicine University of Science and Technology of China Hefei Anhui China; ^4^ Henan Key Laboratory of Genetic and Developmental Disorders Institute of Children's Health, Henan Academy of Medical Sciences Zhengzhou Henan China; ^5^ Research Center for Translational Medicine The Second Affiliated Hospital of Anhui Medical University Hefei Anhui China; ^6^ Department of Cell Biology School of Life Science, Anhui Medical University Hefei Anhui China

1

Dear Editor:

Because aging plays critical roles in immune response and tumour development, alterations of the tumour immune microenvironment with age may be predictive of prognosis. Hence, we analysed single‐cell RNA‐seq (scRNA‐seq) data of gastrointestinal (GI) tract tumours to search for immune cells enriched in elderly patients, and thereby raised an immune index with potential clinical values.

We initially constructed a single‐cell atlas using scRNA‐seq datasets from Gene Expression Omnibus (GEO) for gastric cancer (GC) , colorectal carcinoma (CRC) and oesophageal carcinoma (OSCA), which contained 115 samples and 419 000 cells (Figure S1A,B and Table S1). The lymphoid and myeloid cells were reclustered to subpopulations (Figures S1C–G and S2A,B; Figure 1A,B), and their biased distributions in older (>60 years) and younger (≤60 years) patients were evaluated by the ratio of observed to expected cell numbers (Ro/e) analysis^1^ (Figure 1C). For each subpopulation showing preference for older patients, the top 100 differentially expressed genes were employed to score patients from TCGA datasets via ssGSEA.^2^ The following Kaplan–Meier analysis revealed several subpopulations that were inversely correlated to survival (Figure 1D and Table S2). Further analysis of the differentially expressed genes of these subpopulations discovered multiple commonly enriched pathways (Figure S2C–F). Moreover, among these subpopulations, the CD8T_C2 and Proli_T_C1 had high expression of T cell exhaustion markers (Figure S2G), although this may not necessarily stand for a canonical exhaustion state. Taken together, several immune subpopulations are enriched in older GI tract tumour patients and may affect prognosis.

**FIGURE 1:**
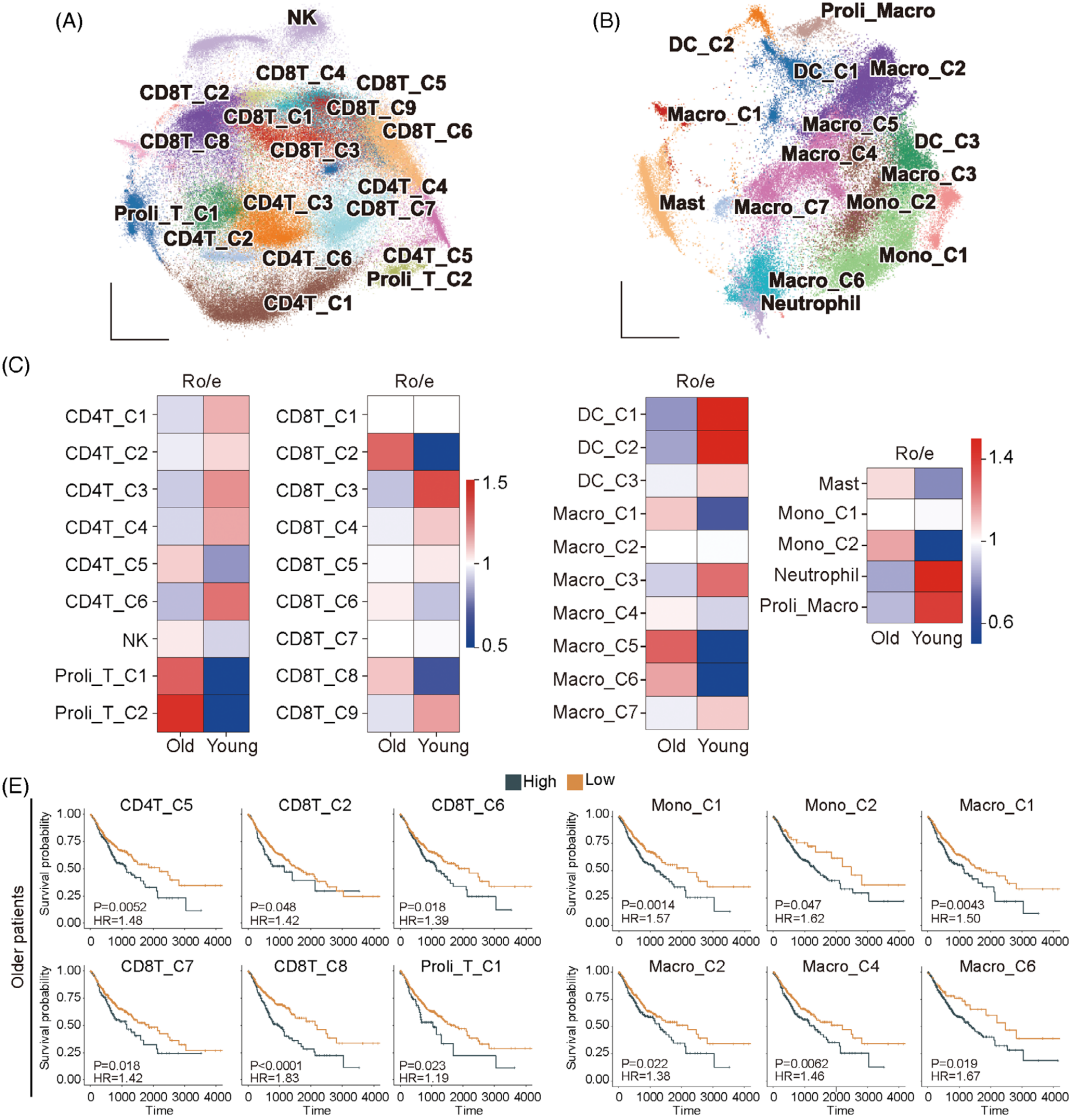
Identification of immune subpopulations with enrichment and clinical implications in older patients. Minimum‐distortion embedding (MDE) visualization of subclusters of lymphoid (A) and myeloid (B) cells in gastrointestinal (GI) tract tumour tissues comprising oesophageal carcinoma (OSCA), colorectal carcinoma (CRC) and GC. Public 10× genomics single‐cell RNA‐seq (scRNA‐seq) Gene Expression Omnibus (GEO) datasets of 419 000 cells from 115 cancer samples were pre‐processed and integrated with R package *Seurat* using classical pipelines, followed by removal of batch effect with the *Harmony* algorithm. Unsupervised clustering was performed based on classical cell markers. The identified T and NK cells were reclustered for lymphoid subpopulations. The identified myeloid cells were reclustered for myeloid subpopulations. (C) Heatmap showing the Ro/e of immune subpopulations enriched in the younger and older patient groups. The subpopulations with Ro/e > 1 were regarded as with preference for an age group. Colour represents Ro/e value. (D) Correlation between immune subpopulations and survivals of older patients with GI tract tumours in the TCGA database. The top 100 differentially expressed genes (DEGs) for the immune subpopulations showing enrichment in older patients were obtained by COSG from scRNA‐seq data and used for scoring these immune cells in each patient of the older group with GSVA. Patients were further stratified into high and low groups followed by Kaplan–Meier analysis using R packages survival and survminer (log‐rank test). The survival curves with statistical significance were shown.

We analysed the potential communications between immune subpopulations with CellChat,^3^, ^4^ and evaluated strength of intercellular signalling pathway with IMPORTANCE (Figure S3A,B). Among the immune subpopulations with enrichment in older patients and correlation to prognosis, nine subpopulations demonstrated markedly altered signalling in older relative to younger patients (ΔIMPORTANCE > 10) (Figure 2A) and had intensive interactions (Figure S3C). Therefore, the nine subpopulations may constitute an elderly enriched immune meta‐cluster (EIM). Deconvolution analysis of spatial transcriptomic data for GC tumours defined distinctive distribution patterns of immune subpopulations, and the pattern with preference for older patients contained several EIM component subpopulations (Figure 2B–D and Figure S3D). The spatial co‐enrichment of EIM component subpopulations were observed by multiple methods (Figure S3D,E). Moreover, GI tract tumour patients in TCGA were scored for EIM followed by Kaplan–Meier analysis, which revealed an inverse correlation between EIM and survival (Figure 2E and Figure S3F). Meanwhile, pathway enrichment network analysis of the signature genes of EIM suggested their participation in immune regulation (Figure S3G). We then stained GI tract tumour sections for molecular markers of EIM. A large proportion of CD8^+^ T cells and CD14^+^/CD16^+^ monocytes belonged to EIM, and more immunofluorescent signals of CD8, CD14, and CD16 were detected in tumours from older relative to younger patients (Figure 2F,G). Besides, while lymphoid subpopulations of EIM scored high for T cell exhaustion markers (Figure S2G), elevated PD1 expression was observed in tumours from older patients (Figure S4A,B). Dual staining of these potential EIM markers showed the same trend (Figure S4C–F). Therefore, the EIM in GI tract tumours is enriched in older patients and inversely correlated with prognosis.

**FIGURE 2:**
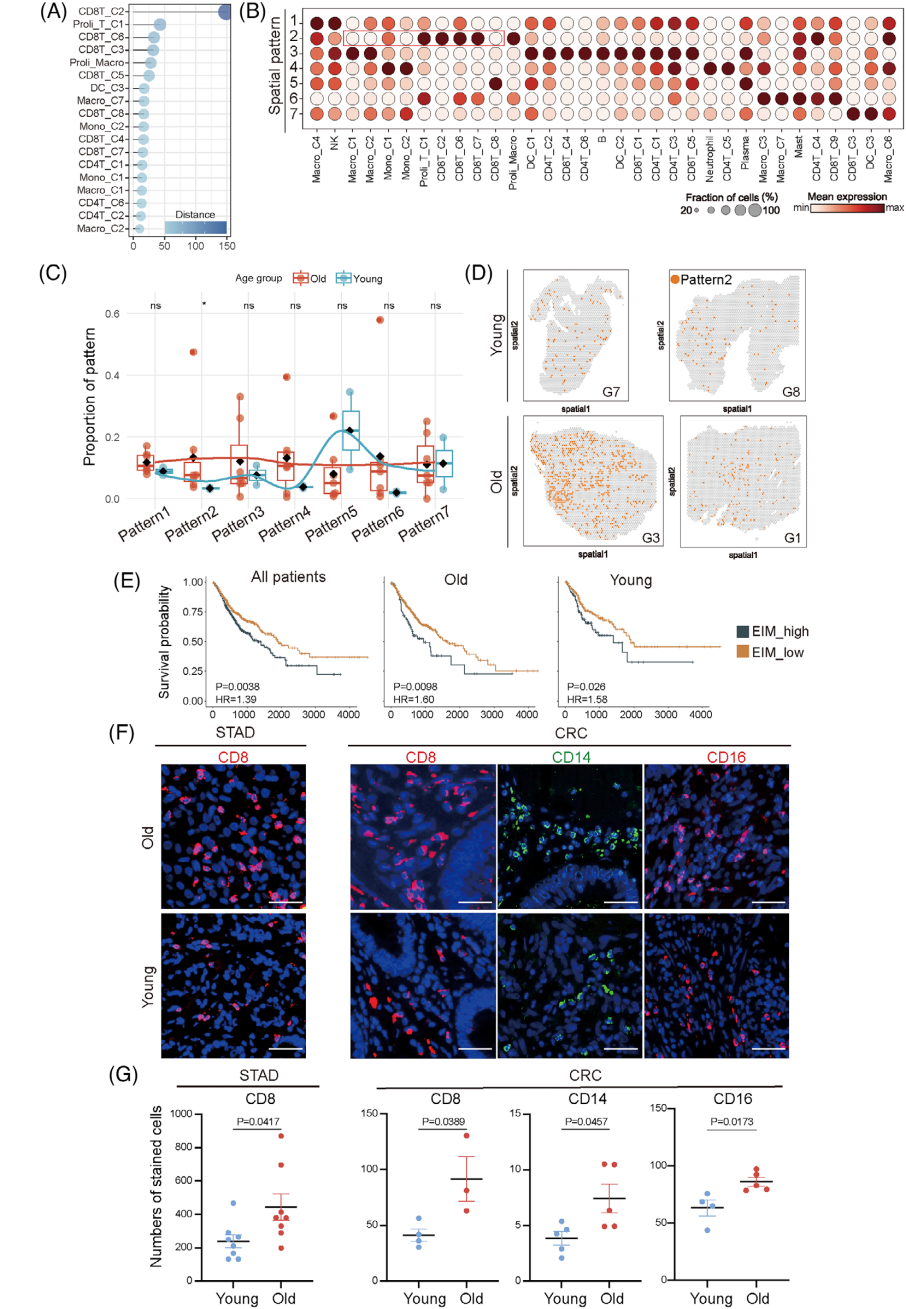
An elder‐enriched immune meta‐cluster (EIM) inversely correlates with patient survival. (A) Alteration of outgoing and incoming intercellular signalling for the indicated immune subpopulations in older relative to younger patients. Alteration of intercellular signalling (distance) was determined by the signalling strength in older patients minus that in younger patients. The immune subpopulations with the most remarkably altered intercellular communications (distance > 10) were shown. Colour bar represents distance. (B) Dot plot showing the abundance of immune subpopulations in the spatial patterns discovered by analysis of public spatial transcriptomic data. Spatial transcriptomic data ofGC(*n* = 9) were analysed with Tangram that integrated the single‐cell RNA‐seq (scRNA‐seq) atlas of gastrointestinal (GI) tract tumour and the spatial transcriptome. The outputs were clustered to spatial patterns by using scanpy. Dot colour represents the abundance of immune subpopulations. The immune subpopulations constituting the EIM were enriched in the spatial pattern 2. (C) Box plot showing the proportions of spatial patterns in spatial transcriptomics data from older and younger patients. The *p* value is calculated by Kruskal test. *p < .05. The spatial pattern 2 showed a preference for older relative to younger patients. (D) Spatial mapping of the spatial pattern 2 in tumours from older and younger GC patients by using Tangram and the public spatial transcriptomic data. A larger proportion of tumour tissues were ascribed to the spatial pattern 2 in older relative to younger patients. (E) Correlation between the EIM and survivals of older patients with GI tract tumours in the TCGA database. The gene signature for the EIM were obtained from scRNA‐seq data and used for calculation of a score for each patient with GSVA. Patients were further stratified into high and low groups followed by Kaplan–Meier analysis using R packages survival and survminer (log‐rank test). The EIM was inversely correlated to patient survival. Representative images (F) and statistical quantifications (G) of immunofluorescent staining of CD8, CD14 and CD16 on human GC and colorectal carcinoma (CRC) samples. Much more positive staining was detected in sections from older relative to younger patients. Scale bar, 30 µm. (GC, *n* = 8 for each age group; CRC, *n* = 3 for older and *n* = 4 for younger group for CD8, *n* = 5 for each age group for CD14, *n* = 5 for older and *n* = 4 for younger group for CD16; mean ± s.e.m.; two‐tailed unpaired *t*‐test).

We applied a machine learning framework composed of four classifiers and tested if EIM could classify elderly patients in TCGA and GEO datasets.^5^, ^6^ The datasets were subjected to every classifier for each immune subpopulation, resulting in area under the curve values as outcomes. The classifier with the highest area under the curve values for a specific dataset was employed (Figure 3A). When it came to classifying elderly patients, the EIM was superior to any subpopulations and outperformed the senescence‐related gene signatures from CellAge^7^ (Figure 3B,C and Figure S5A–C). We utilized scRNA‐seq data of OSCA and CRC tumours to explore the role of EIM in responses to neoadjuvant anti‐PD1‐based immunotherapy (Figure S6A,B). After cell type annotation with our scRNA‐seq atlas, information including age groups and therapeutic outcomes was assigned to cells (Figure 3D and Figure S6C–E). Ro/e analysis of varied distributions showed that most EIM component subpopulations declined in elderly OSCA patients with complete pathologic response relative to those with incomplete pathologic response or major pathologic response (Figure 3E). Consistently, patients with incomplete pathologic response had increased EIM scores than those with major pathologic response or complete pathologic response (Figure 3F). Similar results were obtained in elderly CRC patients showing response or no response to neoadjuvant immunotherapy (Figure S7A–C). As to elderly CRC patients receiving chemotherapy, those with higher EIM scores had worse survivals (Figure 3G and Figure S7D). In summary, EIM is able to predict aging status and response to treatments in elderly patients with GI tract tumours.

**FIGURE 3:**
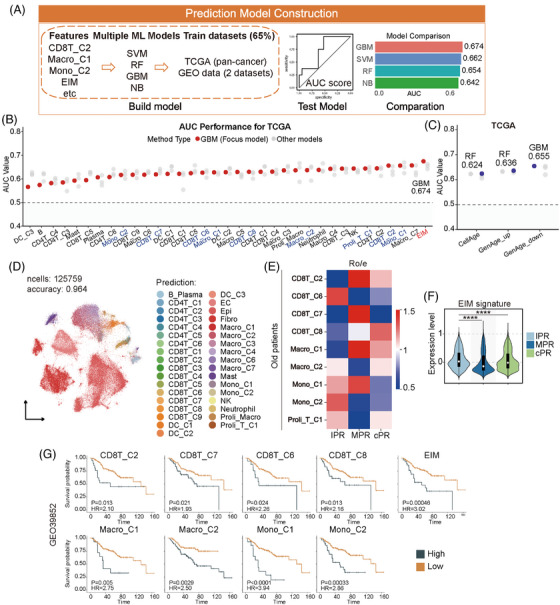
Elder‐enriched immune meta‐cluster (EIM) classifies elderly patients with gastrointestinal (GI) tract tumours and predicts response to treatments. (A) Diagram showing the process of prediction model construction and performance evaluation. The gene signatures of individual component subpopulations (top 100 differentially expressed genes [DEGs], ranked by |log2 fold‐change|) and the EIM (sum of DEGs for component subpopulations) were input to four machine learning classifiers NB, SVM, RF and GBM to construct a model for classifying the elders from patient cohorts. Sixty‐five percent of the gene expression data from the TCGA dataset were used for training, and the remaining TCGA data or the GEO datasets were used for validation. For a specific dataset, the classifier with the best performance as determined by area under the curve (AUC) metrics was finally employed, and AUC values for immune subpopulations in the dataset were assessed. (B) Evaluation of model performance in classifying the elders in TCGA patient cohort. The TCGA (*n* = 940) datasets comprised GC, colorectal carcinoma (CRC), and oesophageal carcinoma (OSCA). The EIM component subpopulations were marked in blue, and EIM was in red. EIM had a better performance than any individual component subpopulations. (C) Evaluation of model performance in classifying the elders using the age‐related gene signatures cellage, geneage_up and geneage_down from the commonly used CellAge database. These age‐related gene signatures had a lower AUC than that of the EIM gene signature. (D) Minimum‐distortion embedding (MDE) visualization of cell clusters in tumours from OSCA patients recruited for neoadjuvant immunotherapy with anti‐PD1 mAb. Public 10× genomics single‐cell RNA‐seq (scRNA‐seq) Gene Expression Omnibus (GEO) datasets for transcriptomes of tumour tissues (125 759 cells, 27 samples) were pre‐processed and integrated with R package *Seurat* using classical pipelines, followed by removal of batch effect with the *Harmony* algorithm. Unsupervised clustering was performed based on classical cell markers. The cell clusters were identified by mapping the cell annotation from the scRNA‐seq atlas of GI tract tumours as reference by using TOSICA. The mapping accuracy was  .964. (E) Heatmap showing the Ro/e of EIM component subpopulations in older patients with different responses to treatment. The subpopulations with Ro/e > 1 were regarded as with preference for a specific group. Colour represents Ro/e value. (F) Violin plot showing expression levels of the EIM gene signature in OSCA patients with different responses to anti‐PD1 mAb neoadjuvant therapy. Each patient was scored for the level of EIM gene signature with GSVA. *p* value is calculated with Wilcox test. ****, *p* < 0.0001. (G) Correlation between the EIM or its component subpopulations and survivals of the older CRC patients treated with adjuvant chemotherapy in GEO datasets. The gene signatures for the EIM or its component subpopulations were used for calculation of a corresponding score for each patient with GSVA. Patients were further stratified into high and low groups followed by Kaplan–Meier analysis using R packages survival and survminer (log‐rank test). The curves with a *p* value < .05 were shown. cPR, complete pathologic response; IPR, incomplete pathologic response; MPR, major pathologic response.

We sought to obtain a prognostic index based on EIM by integrating 10 machine learning algorithms to identify concise gene sets^8^ (Figure 4A). We found that the RSF+Ridge model achieved superior performance and stratified high‐/low‐risk patients across all cohorts (Figure 4A,B). Notably, our EIM‐based model outperformed several classical prognostic signatures in GC and OSCA (Figure 4C). We used the 69 top feature genes from the EIM‐based RSF+Ridge machine learning model to generate an EIM index (Table S3), which was applied to stratify patients into high and low groups. Interestingly, whereas younger patients in the TCGA cohort in general were scored low for the EIM index, a small proportion were ascribed to the high EIM index group and demonstrated poorer prognosis (Figure S7E). The same cut point stratified most older patients into the high EIM index group with worse survival (Figure S7E). However, for all patients together, the EIM index negatived predicted prognosis (Figure 4D). In fact, the EIM index relative to chronological age showed a closer correlation with prognosis (Figure 4E). We further evaluated the transcriptional features of tumours by principal component analysis using the top 3000 highly variable genes of each sample (Figure 4F). Surprisingly, the EIM index, rather than the chronological age, classified patients into distinctive groups in principal component analysis (Figure 4F). Furthermore, patients with higher levels of EIM index had higher expression of the known aging markers such as senescence‐associated secretory phenotype (SASP), regardless of their chronological ages (Figure 4G). Therefore, the EIM index not only predicts prognosis and but also reflects aging status beyond chronological age.

**FIGURE 4:**
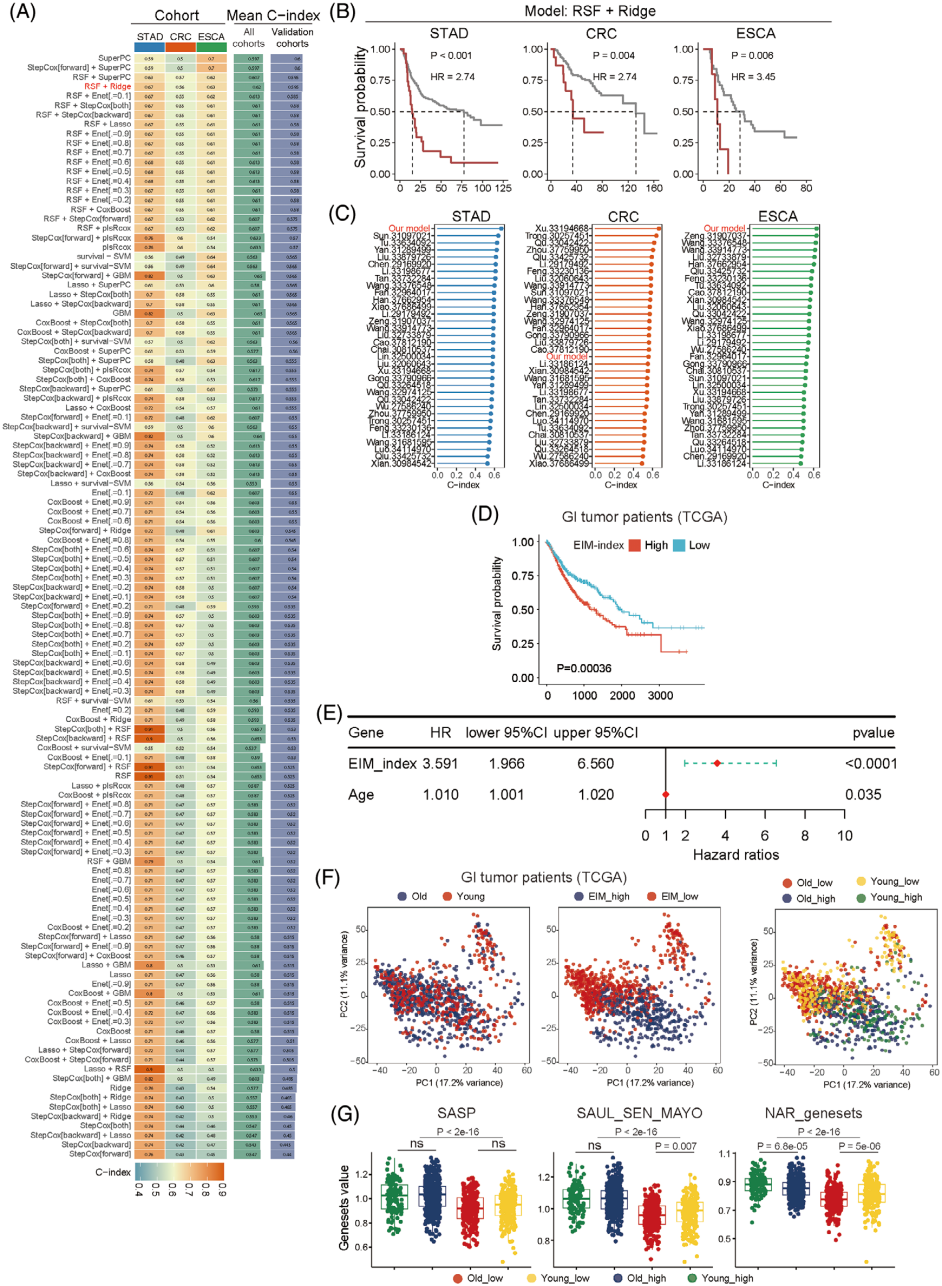
An elder‐enriched immune meta‐cluster (EIM) index predicts prognosis and reflects aging status beyond chronological age. (A) The machine learning–based integration models for prognosis prediction based on transcriptomic data using 10 algorithms. The training datasets were public data of older GC patients treated with adjuvant chemotherapy, and datasets of older colorectal carcinoma (CRC) and oesophageal carcinoma (OSCA) patients were used for validation. The gene signature of EIM were input for predicting patient's prognosis, resulting in 69 genes that constituted the EIM index hereafter. Scale bar represents C‐index for evaluating the models' performance. The RSF+Ridge model achieved superior performance and stratified high‐ and low‐risk patients across all cohorts with a high mean C‐index. (B) Kaplan–Meier survival analysis of older GC, CRC and OSCA patients treated with adjuvant chemotherapy. Public data mentioned above were analysed with the RSF+Ridge model and the EIM index. Kaplan–Meier survival curves were generated using Mime1. (C) Comparison of our model (RSF+Ridge) with published models for prognosis prediction in older GC, CRC and OSCA patients treated with adjuvant chemotherapy by means of C‐index. (D) Kaplan–Meier survival analysis of patients with gastrointestinal (GI) tract tumours in the TCGA database. Each patient was scored for the EIM index using GSVA. Patients were stratified into high and low groups with the threshold of 1.5. (E) Multivariate Cox regression analysis of the overall survival of patients from the TCGA GI tumour cohort. Forest plots with error bars show 2.5% (lower) and 97.5% (higher) bounds of the confidence interval. EIM index of each patient is scored by GSVA. (F) Principal component analysis (PCA) to evaluate the transcriptional features of patients with GI tract tumours. The top 3000 highly variable genes (HVG) for each patient in the TCGA database were obtained for PCA. Each dot represents a patient. Patients of different chronological age groups (left), EIM index scores (middle) or both (right) were depicted. EIM index rather than chronological age classified patients into distinctive groups. (G) Box plots showing the scores of aging‐related genesets in patients of the indicated four groups. Each patient from the TCGA GI tumour cohort was scored for the indicated genesets of aging markers with GSVA, and patients were stratified with 1.5 as the cut‐off score for the EIM index. All *p* values are calculated by Wilcox test. ns, *p* > .05.

People just begin to utilize single‐cell analysis to study the immune features associated with age.^9^ In this study, through scRNA‐seq analysis of immune landscapes in GI tract tumours from older and younger patients, we discovered the EIM with enrichment in the elders and inverse correlation with patient survivals, and consequently raised the EIM index with potential prognostic values. Our findings suggest that alterations of the tumour immune microenvironment in aged patients may be quantified to foresee tumour development, although the causes of such shifts require further studies.

## 2 AUTHOR CONTRIBUTIONS

F.L., J.C. and J.W. contributed equally to this work. W.Z., H.W., A.Z. and D. L. developed the working hypothesis and scientific concept. W.Z., F.L., J. C. and J.W. designed the experiments, analysed the data and prepared the manuscript. F.L. and J.C. performed the experiments and organized the data. D.L. and X.L. provided surgical specimens. All authors assisted the experiments, helped manuscript preparation and provided scientific input.

## 4 CONFLICT OF INTEREST STATEMENT

The authors declare no conflicts of interest.

## 6 ETHICS STATEMENT

Written informed consent was obtained from all participants, and the related experiments were approved by the Medical Research Ethics Committee of The First Affiliated Hospital of the University of USTC with the permit no. 2023KY454. Ethics approval of use of the public sequencing data from human patients was exempted by the Medical Research Ethics Committee of The First Affiliated Hospital of the University of USTC.

## Supporting information



Supporting Information

## Data Availability

All the sequencing data are publicly available. Details are provided in Supporting Information. Codes used in this study are available upon request.
