# Supplementary material for: Alterations of the immune microenvironment with age predicts patient prognosis of gastrointestinal tract tumours
Source: Clin Transl Med. 2026 Jan 7;16(1):e70592. doi: 10.1002/ctm2.70592 (PMC12779932; doi:10.1002/ctm2.70592)
Supplement: Supplementary file 1 — Supporting Information [file CTM2-16-e70592-s001.docx]

**Methods**

**scRNA-seq data collection and preprocessing**

Transcriptome data of 115 patients with STAD, CRC and ESCA were obtained from scRNA-seq datasets that utilized 10× platform. Most datasets were derived from whole tumor tissues. Specifically, datasets for STAD (GSE183904, GSE163558, GSE234129 and GSE150290), COAD (GSE108989, GSE132465 and GSE146771) and ESCA (PRJNA606979 (NIH) and PRJCA016745 (OMIX)) were used for analysis.

After obtaining the original scRNA-seq data, output folders of cellranger were loaded in the *Read10X* function of the Seurat (v.4.3.0) package, and count matrices were loaded in the *fread* function. Subsequently, the *merge* function was applied to integrate all patient data into a unified compilation object along with unique barcode labels. After then, cells with fewer than 200 detected genes or with more than 40% mitochondrial content out of total detected genes were discarded. The omicverse package was employed for data normalization, resulting in identification of 3,000 highly variable genes. Principal component analysis (PCA) was then conducted for dimensionality reduction through incorporating the highly variable genes, and the top 30 principal components (PCs) were selected for subsequent analysis. The harmony (v.1.0.3) was utilized to mitigate batch effects. Unsupervised clustering was conducted by omicverse function of harmony, and the identified clusters were visualized using mde visualization under default settings.

Multiple rounds of clustering were performed to get an accurate subgroup annotation. Initially, major cell types such as CD4^+^ T cells, CD8^+^ T cells, myeloid cells, fibroblasts, etc., were identified using scanpy package and the *COSG_markers* function of the COSG R package that provided subgroup-specific genes. To further zoom in and identify immune subsets, non-immune cell populations were discarded, and additional clustering analysis was performed on larger immune subpopulations followed by identification of the top 50 differentially expressed genes using the aforementioned methods.

**Pathway enrichment analysis**

Gene set enrichment analysis was performed using AUCell and UCell (v2.2.0) to quantify transcriptional signatures in single-cell RNA sequencing (scRNA-seq) datasets processed through Seurat or Scanpy. To evaluate exhaustion status among T cell subsets, we computed scores for exhaustion markers using the AddModuleScore_UCell function. The scores, representing the mean enrichment of curated gene sets, were statistically compared across cell populations and visualized as boxplots to delineate transcriptional differences between functional states.

To delineate the mechanistic role of age-enriched immune subpopulations, cluster-specific signature genes were identified as differentially expressed genes (DEGs) meeting statistical thresholds (log2 fold-change >1.2, adjusted p < 0.05). Functional enrichment analysis was conducted using these cluster-specific DEGs across all subpopulations via ​SCP, followed by the construction of pathway interaction networks to resolve coordinated biological processes. To enhance visualization of age-associated functional states, the ​EnrichmentPlot​ function from SCP was applied with default 'network' parameters.

**Ro/e analysis**

To assess age-associated differences in immune cell distribution, we quantified the relative abundance of immune subpopulations in tumors from older versus younger patients using the ratio of observed to expected cell frequencies (Ro/e). The expected cell numbers for each subpopulation in patients of the two age groups were obtained from the chi-square test. Subpopulations were classified as significantly enriched within a specific age cohort if their Ro/e values exceeded 1, indicating a higher-than-expected prevalence relative to the baseline distribution.

**Gene set variation analysis**

To assign pathway activity, we first get top100 DEGs for each immune subpopulation. Then, the enrichment score for each immune subpopulation was calculated with the GSVA (v.1.46.0) package based on the corresponding signature gene set that comprises the top markers obtained with the COSG (v.0.9.0) package.

**TCGA RNA-seq data analysis**

***Data collection***

The expression data and clinical information of Genomic Data Commons (GDC) TCGA were downloaded from UCSC Xena project (http://xena.ucsc.edu). Samples were scored by using *GSVA*, which reflected the relative abundance of indicated cell types. Gene signatures for cell populations were inferred from their DEGs by *COSG*. For a given type of cancer, the same dataset of bulk RNA-seq was used for bioinformatic analysis throughout the study. Gene set enrichment analysis for TCGA data are using GSVA package.

***Survival analysis***

The bulk RNA-seq data underwent preprocessing to log2(FPKM+1) and subsequent analysis and visualization using the survival (v.3.5) and survminer (v.0.4.9) packages. Kaplan-Meier method was employed to summarize overall survival for each group. Optimal cut points for variables were determined through group comparisons based on GSVA score, with the functions *surv_cutpoint* and *surv_categorize* utilized to calculate appropriate groupings for each variable. Group comparisons were then conducted using the log-rank test. Then, R package survival and survminer were utilized to do the analysis and generate the curves representing the difference between two clusters.

**CellChat analysis of cell-cell interactions**

For cell-cell interactions between immune populations, CellChat (v.2.1.1) was performed on two Seurat objects derived from older and younger patients, respectively, and the preprocessed CellChat object was obtained with the function *mergeCellChat*. The different strength of interactions in the cell-cell interaction network between the two datasets were visualized with circle plot. Then the up-regulated and down-regulated signaling axes between immune populations in older and younger tumor patients were determined. The interaction strength between the paired cell subsets was calculated according to the gene expression of the signaling axes. The statistical significance of the increase in signaling axes was determined with permutation test, and those axes with a p value < 0.05 were considered significantly increased.

**Collection of spatial transcriptomics data**Three published spatial transcriptomics datasets for STAD (GSE251950) were downloaded. Data were integrated and processed by Seurat. The spatial data were subjected to normalization using *SCTransform*. We assessed gene expression levels and ratio of mitochondrial genes in every spot but did not filter any spot in the tumor tissues to maintain the integrity of the sections.

**Spatial deconvolution with *Tangram***

For the spatial distributions of the cell populations identified by the analysis of the scRNA-seq atlas, *Tangram* was employed to integrate the scRNA-seq data with the 10× Genomics Visium mRNA count matrix. Briefly, the *Tangram* model back-convolves mRNA counts from 10× Visium data using transcriptional signatures of a reference cell type to estimate the abundance of different cell populations at each spatial spot. This model is implemented through the *Tangram* function in the omicverse on python. We used the atlas as the reference single-cell data that included the cell populations of interest. We then identified highly variable genes that were common between the reference single-cell data and the spatial transcriptomics data, which were used for model training. Second, the *cells* model was selected to infer the proportions of various cell types in each spot of the spatial transcriptomics data. For training, the model was run for more than 500 epochs until the stabilization of performance scores.

The outputs of Tangram provided the abundance of all cell types in each spatial spot, but lacked proper overviews of tumor sections. Therefore, we clustered the outputs using the *sc.pp.neighbors* and *sc.tl.leiden* functions in *Scanpy* to identify spatial structural domains based on the proportions of cell subpopulations. These structural domains were then merged based on the similarity of the expression abundance of cell populations within each domain, ultimately defining the spatial patterns.

**Construction of clinical predictive models**

To develop clinically predictive models of age for tumor patients, we trained machine learning classifiers—including naive Bayes (NB), support vector machine (SVM), random forest (RF) and gradient boost machine (GBM)—using immune subpopulation enrichment scores. Model training was performed on TCGA cohorts including STAD, CRC and ESCA, with gene sets representing each immune subpopulation defined by the top 100 DEGs as previously described. Validation was conducted on independent colorectal cancer datasets (GSE39582 and GSE17538) to assess generalizability. All analyses were implemented via the ​naivebayes​, gbm, randomForest and caret R package, optimizing parameters for cross-cohort reproducibility.

**Single-cell analysis of data from patients treated with immunotherapy**

To interrogate immune cell type redistribution under immune checkpoint inhibition (ICI), we analyzed scRNA-seq data from CRC (GSE20556) and ESCA (PRJCA016745, OMIX) cohorts generated via the 10× Genomics Chromium platform. Cohorts comprised patients stratified by PD-1 inhibitor treatment status (treated vs. treatment-naive) and annotated with objective response information (respond level).

For cross-cohort cell type annotation consistency, we used ​TOSIC, a transformer-based framework from the ​omicverse​ ecosystem. Reference datasets pre-annotated with cell type metadata were used to train TOSICA, while query datasets included preprocessed scRNA-seq profiles from PD-1-treated samples. Default model parameters were initialized and trained.

**Machine learning algorithms construction by Mime1**

Prognostic models were constructed using the ​Mime1​ computational framework. The ML.Dev.Prog.Sig ​function was employed to train EIM gene signatures on discovery cohorts, followed by multi-dataset validation to assess generalizability. Model performance was quantified via concordance index (C-index) distributions and Kaplan-Meier survival analysis. To benchmark clinical utility, we computed risk scores for published prognostic signatures (e.g., vascular angiogenesis, m6A-related models from Mime1) and performed C-index comparisons with our model.

To evaluate transcriptional similarity among tumor patients, we employed principal component analysis (PCA) with the R package stats on expression matrices comprising the top 3,000 most variably expressed genes. Each dot in PCA represents a patient that belongs to an age group and is scored for EIM index. The same score of EIM index was utilized to stratify patients into high and low groups for survival analysis and PCA.

**Human tumor specimens**

De-identified tumor specimens were collected from the First Affiliated Hospital of the University of Science and Technology (Anhui, China) in accordance with an Institutional Research Ethics Committee-approved protocol. Informed consent was obtained from all subjects and experiments were approved by the Medical Research Ethics Committee of The First Affiliated Hospital of the University of Science and Technology of China. Participants are not compensated for the involvement in this study. Sex is not considered in the study design. STAD samples were from younger patients aged 37-60 years and older patients aged 66-83 years; 22.5% of the younger patients and 32.5% of the older patients were female. COAD samples were from younger patients aged 25-59 years and older patients aged 67-91 years; 41.3% of the younger patients and 38.1% of the older patients were female.

**Immunofluorescent staining**

For immunofluorescent staining, formalin-fixed paraffin-embedded (FFPE) sections at a thickness of 3 microns were rehydrated. Antigen retrieval was performed by boiling the sections in Tris/EDTA buffer pH 9.0 for 10 minutes. Sections were then incubated in a PBS solution containing 2% BSA plus 0.3% Triton X-100 for 1 hour at room temperature for blocking and permeabilization. Sections were incubated with primary antibodies in PBS containing 2% BSA overnight at room temperature. After then, the sections were washed with PBS and incubated with secondary antibodies (1:500 dilution) plus Hoechst (1:20,000 dilution) in PBS containing 2% BSA for 2 hours at room temperature in dark. After final wash with PBS, a coverslip was mount on sections, and the staining was subjected to microscopy. Specific antibodies against CD8 (1:10, ZA-0508, ZSGB-BIO), PD1 (1:10, IM362, LBP), CD14 (1:10, ZA-0532, ZSGB-BIO), CD16 (1:200, 66779-1-Ig, Proteintech) were used for the staining as indicated. The numbers of cells with positive staining were counted by ImageJ.

**Statistics and reproducibility**

Data were analyzed with Microsoft Excel functions, Prism 9 software (GraphPad) or the abovementioned R programming language. Analyses were performed using Python and R.

Statistical analyses were performed as described in legends. Pearson correlation was used to estimate correlations among immune cell subsets. Statistical significance was determined by Kruskal-Wallis test, Wilcoxon test, or t test.

**Supplementary Tables**

| **Characteristics** | **Categories** | **Numbers** |
| --- | --- | --- |
| Total patients |  | 115 |
| Gender | Male | 58 (50.43%) |
|  | Female | 42 (36.52%) |
|  | NA | 15 (13.05%) |
| Age group | Young | 41 (35.65%) |
|  | Old | 74 (64.35%) |
| Tumor type | STAD | 41 (35.65%) |
|  | CRC | 48 (41.74%) |
|  | ESCA | 26 (22.61%) |

**Table S1. Information of the samples in the scRNA-seq atlas.**

| **Cluster** | **K-M p value (survcutpoint)** | **K-M p value (50% cut-off)** | **Cox p value** | **Old/young preference** |
| --- | --- | --- | --- | --- |
| B | 0.14 | 0.66 | 0.184882997276876 | NA |
| CD4T_C1 | 0.21 | 0.68 | 0.554508131041542 | young |
| CD4T_C2 | 0.035 | 0.92 | 0.506643495403999 | young |
| CD4T_C3 | 0.0019 | 0.54 | 0.386245836777945 | young |
| CD4T_C4 | 0.0015 | 0.12 | 0.0258721366559438 | young |
| CD4T_C5 | 0.0052 | 0.097 | 0.0182491894924986 | old |
| CD4T_C6 | 0.011 | 0.77 | 0.381108715499159 | young |
| CD8T_C1 | 0.15 | 0.87 | 0.42434984026918 | old |
| CD8T_C2 | 0.048 | 0.7 | 0.754318598458694 | old |
| CD8T_C3 | 0.0067 | 0.048 | 0.0317626168765188 | young |
| CD8T_C4 | 0.053 | 0.72 | 0.316405508925256 | young |
| CD8T_C5 | 0.075 | 0.35 | 0.133084614685585 | young |
| CD8T_C6 | 0.018 | 0.13 | 0.0888480817025582 | old |
| CD8T_C7 | 0.018 | 0.16 | 0.036779152310653 | old |
| CD8T_C8 | 0.0001 | 0.0016 | 0.000393618625193708 | old |
| CD8T_C9 | 0.0049 | 0.24 | 0.0399758386517231 | young |
| DC_C1 | 0.051 | 0.37 | 0.164748157420075 | young |
| DC_C2 | 0.014 | 0.097 | 0.0222259839652684 | young |
| DC_C3 | 0.024 | 0.096 | 0.110885201265523 | young |
| Macro_C1 | 0.0043 | 0.036 | 0.0263636062784972 | old |
| Macro_C2 | 0.022 | 0.04 | 0.0588961831193234 | old |
| Macro_C3 | 0.0029 | 0.11 | 0.067463272663154 | young |
| Macro_C4 | 0.0062 | 0.024 | 0.034802523989673 | old |
| Macro_C5 | 0.079 | 0.52 | 0.272429941681538 | old |
| Macro_C6 | 0.019 | 0.084 | 0.0342541671063893 | old |
| Macro_C7 | 0.0029 | 0.0073 | 0.0144813464154694 | young |
| Mast | 0.06 | 0.91 | 0.0813514145670772 | old |
| Mono_C1 | 0.0014 | 0.0053 | 0.0317848388124688 | old |
| Mono_C2 | 0.047 | 0.18 | 0.0585719520466227 | old |
| NK | 0.11 | 0.59 | 0.3765201122735 | old |
| Neutrophil | 0.0054 | 0.062 | 0.100861719066232 | young |
| Plasma | 0.046 | 0.76 | 0.1418933361494 | NA |
| Proli_Macro | 0.032 | 0.79 | 0.302482438909476 | young |
| Proli_T_C1 | 0.023 | 0.55 | 0.690414166765308 | old |
| Proli_T_C2* | N/A | N/A | N/A | old |
| * Less than 0.5% in total immune cells and omitted from further analyses | | | | |

**Table S2. P values of survival analyses for the immune subpopulations.**

| **EIM_index** |  |
| --- | --- |
| FCGR2A | DUSP4 |
| FOLR2 | RNF125 |
| MS4A6A | VPS37B |
| CSF1R | GNG2 |
| DAB2 | ZNF331 |
| GPR34 | CTSE |
| CD14 | GZMK |
| CD209 | SH3BGRL3 |
| MS4A7 | HLA.C |
| F13A1 | PSMB9 |
| ABCA1 | RPS23 |
| CXCL16 | RORA |
| SLC8A1 | ZFP36 |
| EPB41L3 | ATXN1 |
| COLEC12 | EIF1 |
| NFE2 | TYMS |
| CYP1B1 | RRM2 |
| RBP7 | ASPM |
| AP1S2 | MKI67 |
| PTX3 | NUSAP1 |
| STXBP2 | TPX2 |
| CD93 | TOP2A |
| TIMP1 | ATAD2 |
| GPBAR1 | PTTG1 |
| LYN | LRRN3 |
| RASGRP2 | KIR2DL4 |
| WARS | GTDC1 |
| SHC4 | CCNB1 |
| TSHZ2 | SLC9A9 |
| SETBP1 | ITGA1 |
| LRBA | TFF1 |
| ZSWIM6 | TFF2 |
| OMD | SULT1C2 |
| ST6GALNAC3 | CXCR4 |
| ZBED2 |  |

**Table S3. Genes constitute the EIM index**

**Supplementary Figures**

**
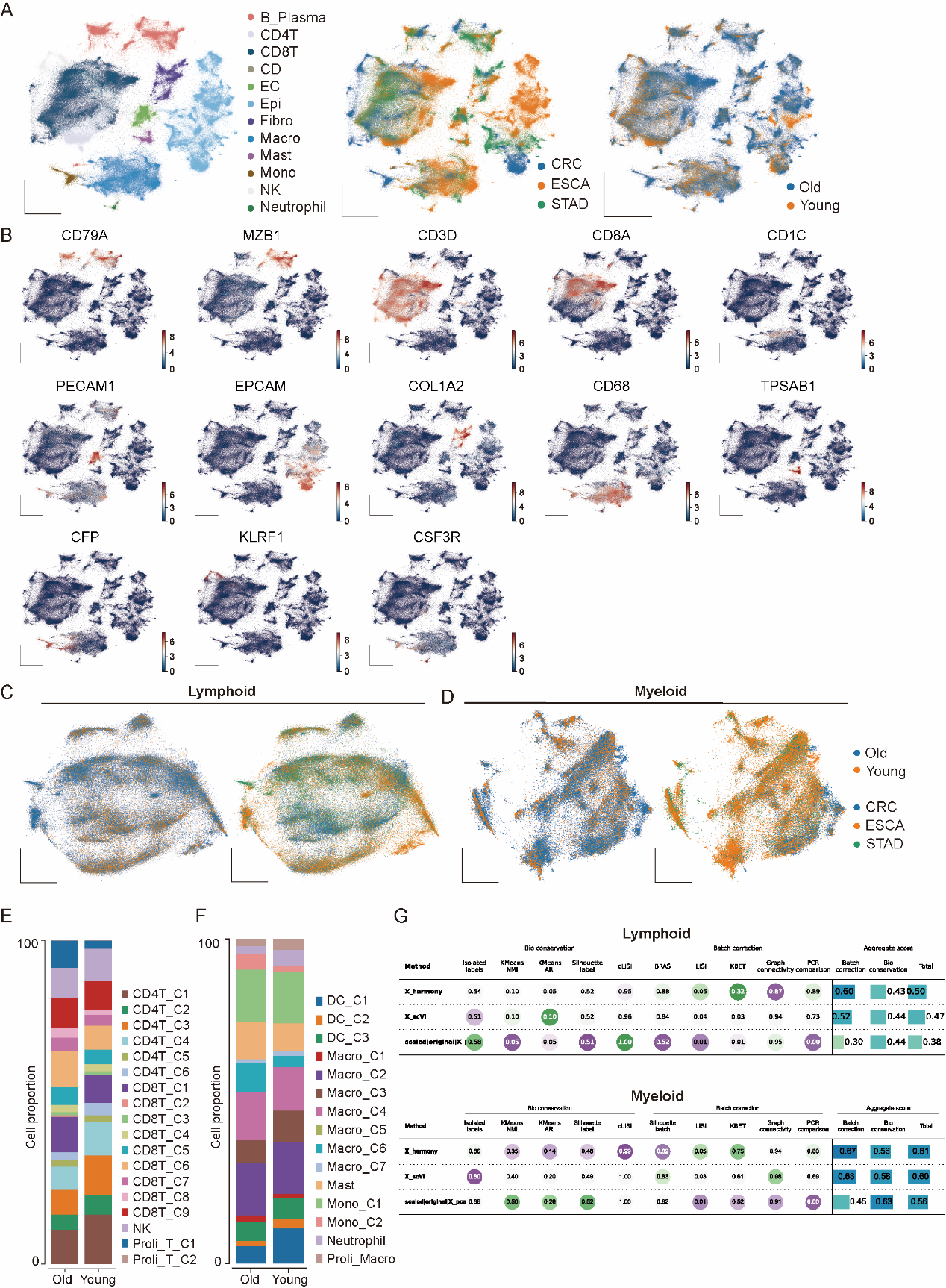
**

**Fig. S1.** **Construction of a GI tract tumor atlas using public scRNA-seq datasets**

1. Minimum-Distortion Embedding (MDE) visualization of cell clusters in GI tract tumor tissues comprising ESCA, CRC and STAD. Public 10× Genomics scRNA-Seq GEO datasets of 419,000 cells from 115 cancer samples were preprocessed and integrated with R package *Seurat* using classical pipelines, followed by removal of batch effect with the *Harmony* algorithm. Unsupervised clustering was performed based on classical cell markers. The cells from different types of tumors were depicted in the middle. The cells belonging to older and younger patients were depicted on the right.
2. Visualization of classical cell markers in the scRNA-seq atlas constructed above. cell clusters in GI tract tumor tissues comprising ESCA, CRC and STAD. These markers defined meta-clusters including B/plasma cells (marked by CD79A and MZB1), CD4 T cells (marked by CD3D), CD8 T cells (marked by CD3D and CD8A), dendritic cells (DC) (marked by CD1C), endothelial cells (EC) (marked by PECAM1), fibroblasts (marked by COL1A2), macrophage (marked by CD68), mast cells (marked by TPSAB1), monocyte (marker by CFP), natural killer (NK) cells (marked by KLRF1), and neutrophil (marked by CSF3R).

**C** and **D**. Visualization of lymphoid (**C**) and myeloid (**D**) subclusters in the scRNA-seq atlas constructed above. The source tumor types and the age groups were depicted.

**E** and **F**. Bar graphics showing the proportions of lymphoid (**E**) and myeloid (**F**) subclusters in older and younger patients in the scRNA-seq atlas.

**G**. Evaluation of the outcome of batch effect removal with harmony and scVI algorithms in lymphoid (upper) and myeloid cells (lower) by using the scib function from python package omicverse. Processing with harmony demonstrated a better batch correction and biological conservation, and the resultant data was used for onward analyses. PCA stands for no batch effect removal.


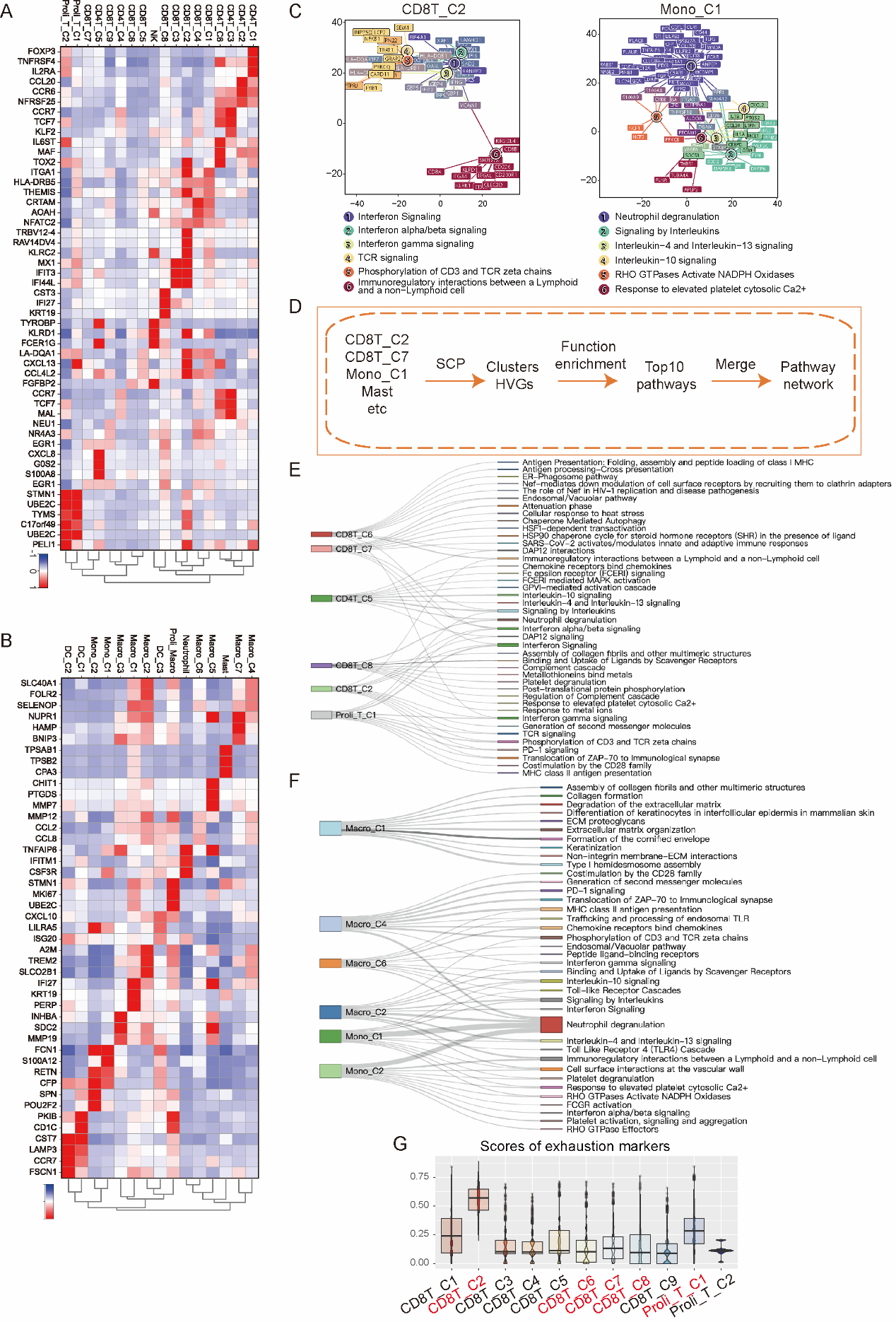


**Fig. S2. Pathway enrichment analysis of immune subpopulations enriched in older patients with GI tract tumors**

**A** and **B**. Panels of the differentially expressed genes with high expression in the indicated lymphoid (**A**) and myeloid (**B**) subpopulations. Each cluster was automatically marked by three specific genes with high expression. Color stands for the mean expression of markers in the subpopulation.

**C**. Visualization of pathway enrichment networks of lymphoid (left) and myeloid (right) subpopulations with preference for older patients using SCP. The immune subpopulations that were enriched in older patients and inversely correlated to survival were subjected to analysis. CD8T_C2 and Mono_C1 were shown as examples for lymphoid and myeloid cells. Highly variable genes (log2FoldChange > 1.2 and p_adj < 0.05) of a given subpopulations were inputted for REACTOME enrichment by using clusterprofiler. The resultant pathways were clustered.

**D**. Diagram showing the obtainment and visualization of pathway enrichment networks of immune subpopulations enriched in older patients. Highly variable genes (log2FoldChange > 1.2 and p_adj < 0.05) of each immune subpopulation were used to obtain the top10 enriched pathways with functions in immunology and cell biology. The resultant pathways were clustered for lymphoid and myeloid subpopulations, respectively.

**E** and **F**. Visualization of pathway enrichment networks of lymphoid (**E**) and myeloid (**F**) subpopulations enriched in older patients with potential clinical implications. Highly variable genes (log2FoldChange > 1.2 and p_adj < 0.05) of each subpopulation were inputted for Reactome enrichment by using clusterprofiler. Top10 pathways for each subpopulation were clustered as network and visualized with SCP.

**G**. Boxplot showing the scores of exhaustion markers in the indicated T cells. Expression of exhaustion markers in all the CD8^+^ T and proliferating T subpopulations was evaluated by analysis of the scRNA-seq data from older and younger patients with UCell. Y axis stands for score of exhaustion. The subpopulations enriched in older patients were labelled in red. The exhaustion markers include PDCD1, TOX2, TIGIT, TNFRSF4, CXCL13, TNFRSF9, TOX, CXCL13, HAVCR2, and LAG3. The CD8T_C2 and Proli_T_C1 had the highest expression of exhaustion markers.


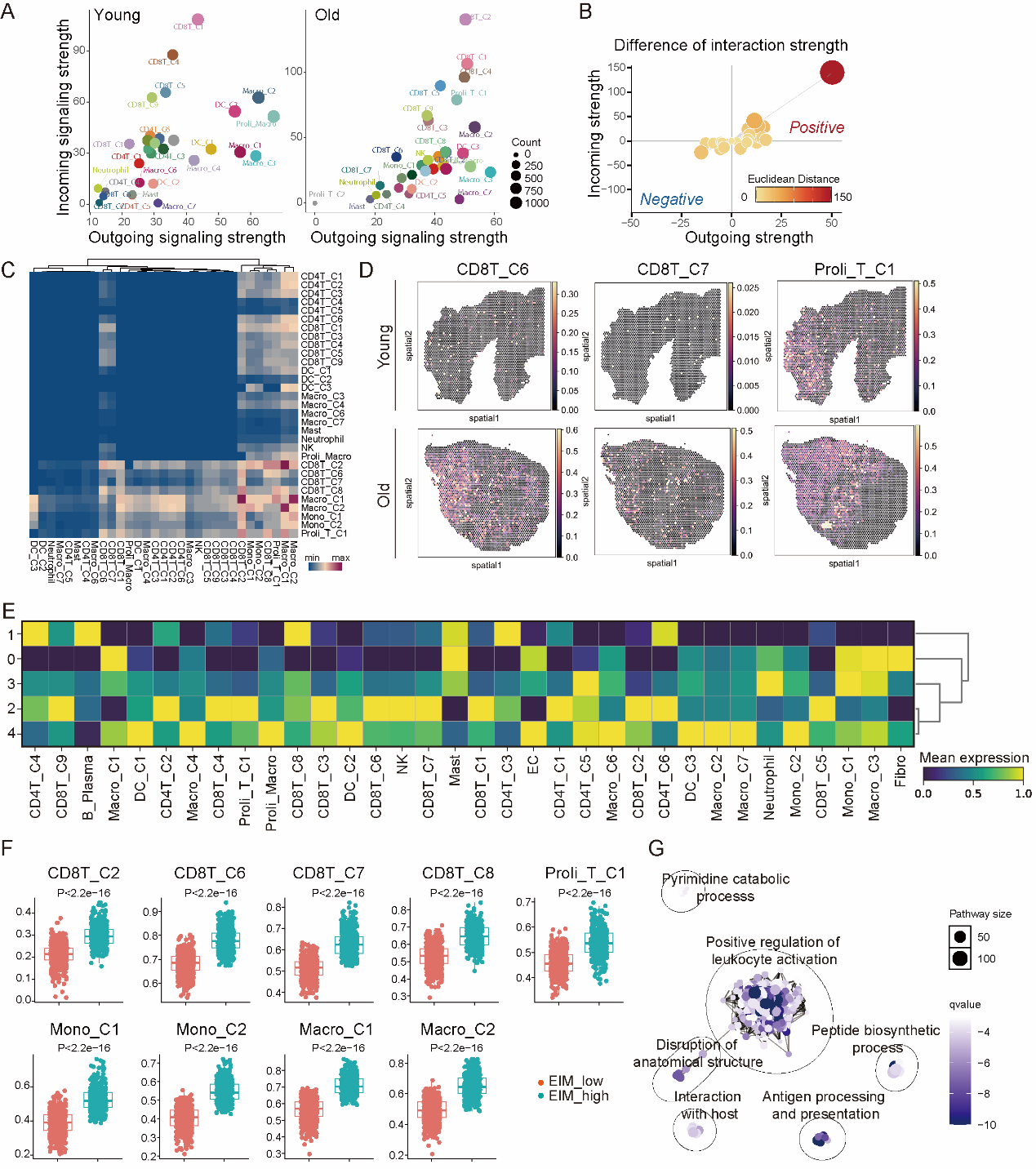


**Fig. S3. An elder-enriched immune meta-cluster (EIM) inversely correlates with patient survival**

**A**. Strength of outgoing and incoming intercellular signaling for the indicated immune subpopulations in older (right) and younger (left) patient cohorts. The strength of intercellular signaling was evaluated with the CellChat package. Dot size reflects numbers of intercellular interactions.

**B.** Alteration of outgoing and incoming intercellular signaling for immune subpopulations in older relative to younger patients. Alteration of intercellular signaling (distance) was determined by the signaling strength in older patients minus that in younger patients. Color bar represents distance.

**C.** Heatmap showing the intercellular communications from the immune subpopulations enriched in the older patients to all immune subpopulations. Immune subpopulations with enrichment in older patients, inverse correlation with survival, and altered intercellular communications (distance >10) were picked up as the sender cells for the analysis of intercellular communications. Color bar represents interaction strength.

**D.** Spatial distributions of the indicated immune subpopulations enriched in the spatial pattern 2 in patients of different age groups using Tangram. Color bar represents the abundance of immune subpopulations. The CD8T_C6, CD8T_C7, and ProliT_C1 subpopulations not only had similar spatial distributions, but also had higher abundance in older relative to younger patients.

**E**. Heatmap showing the abundance of different cell populations in spatial niches as discovered by the GraphST algorithm. Several EIM component populations (Proli_T_C1, CD8T_C2, CD8T_C6, CD8T_C8, Mono_C1, and Mono_C2) were enriched in the niche #2.

**F.** Box plots showing the abundance of EIM component subpopulations in patients with high and low EIM scores. The gene signature for a given component subpopulation was obtained from scRNA-seq data and used for calculation of a corresponding score for each patient with GSVA. The p value was calculated by Wilcoxon test.

**G**. Visualization of pathway enrichment networks derived from signature genes of EIM populations. DEGs of EIM were input for GO enrichment using *clusterprofiler*. The resultant pathways with statistical significance were clustered for automatic generation of the enrichment networks using *aPEAR*. Dot color represents qvalue. Dot size represents the pathway size. EIM may have a close relationship with pathways involved in immune regulation


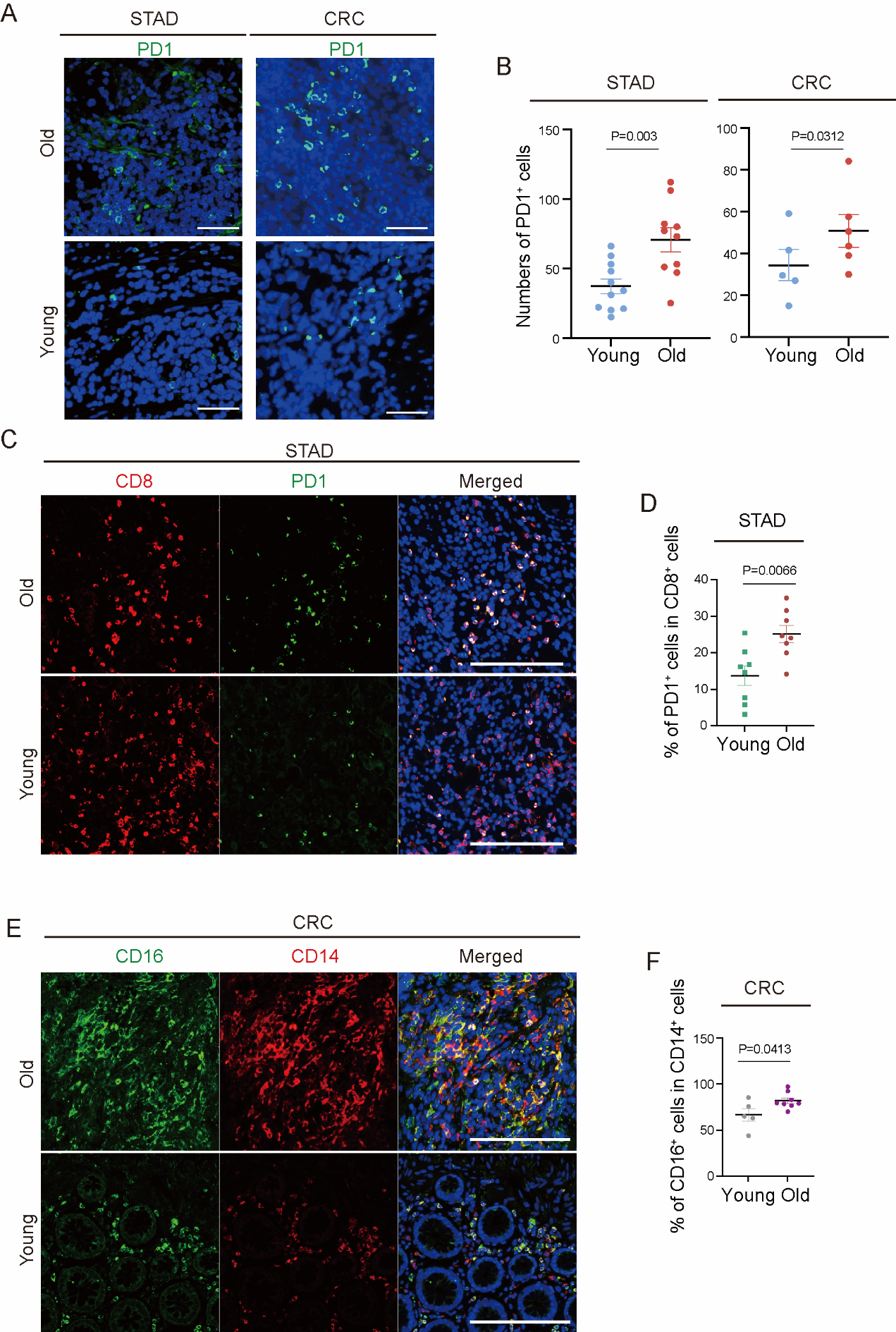


**Fig. S4. Molecular markers of EIM have stronger staining in older patients**

**A** and **B**. Representative images (**A**) and statistical quantifications (**B**) of immunofluorescent staining of PD1 on human STAD and CRC samples. Much more PD1 signals were detected in sections from older relative to younger patients. Scale bar, 30 μm. (STAD, n = 10 for older and n = 11 for younger group; CRC, n = 6 for older and n = 5 for younger group; mean ± s.e.m.; two-tailed unpaired t-test)

**C** and **D**. Representative images (**C**) and statistical quantification (**D**) of immunofluorescent staining of PD1 and CD8 on human STAD samples. A larger proportion of CD8 cells were stained with PD1 in sections from older relative to younger patients. Scale bar, 30 μm. (n = 8 for older and n = 8 for younger group; mean ± s.e.m.; two-tailed unpaired t-test)

**E** and **F**. Representative images (**E**) and statistical quantification (**F**) of immunofluorescent staining of CD14 and CD16 on human CRC samples. A larger proportion of CD14 cells were stained with CD16 in sections from older relative to younger patients. Scale bar, 30 μm. (n = 8 for older and n = 5 for younger group; mean ± s.e.m.; two-tailed unpaired t-test)


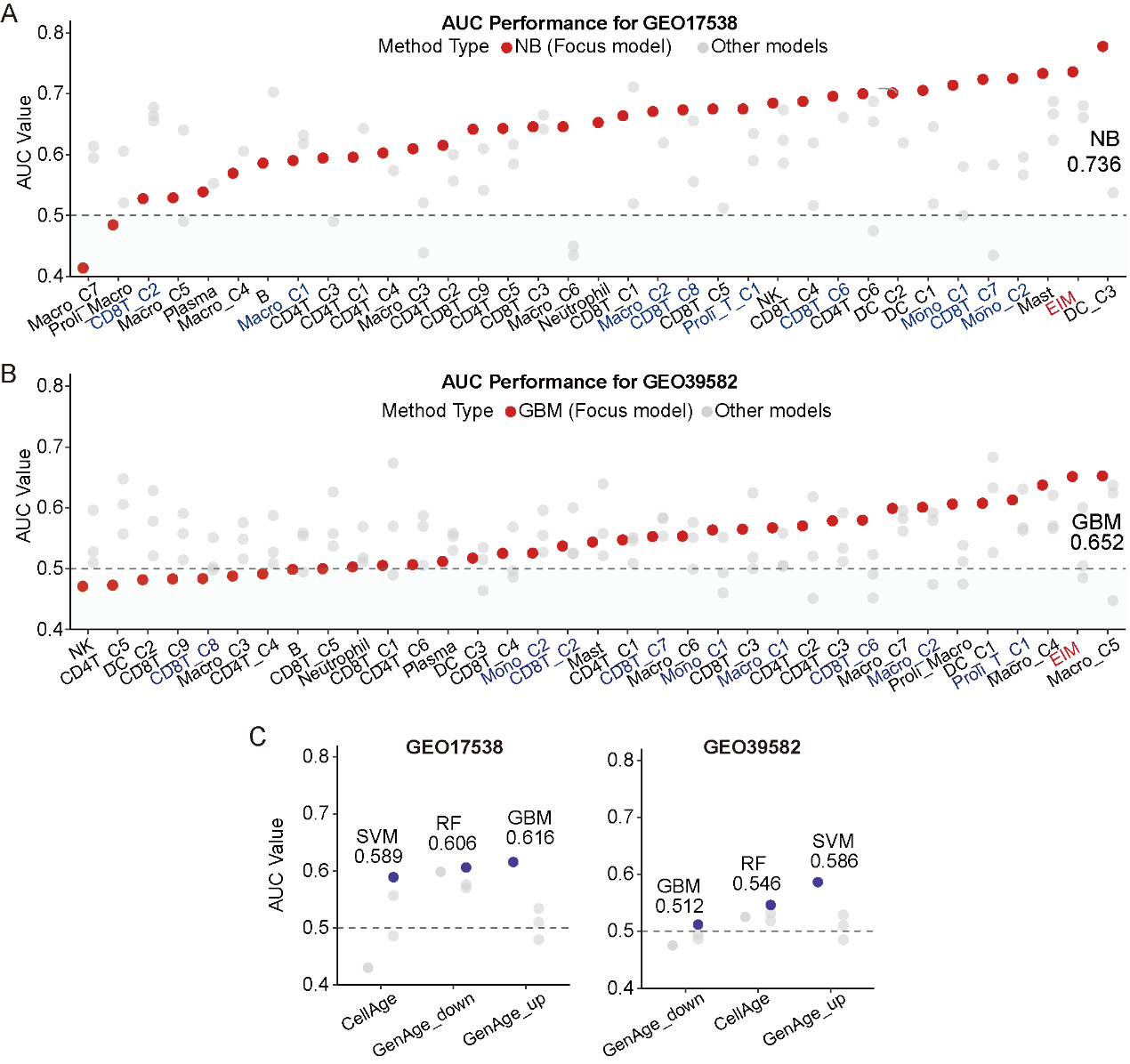


**Fig. S5. EIM in whole classifies elderly patients with GI tract tumors**

**A and B.** Evaluation of model performance in classifying the elders in GSE17538 (**A**), and GSE39582 (**B**) patient cohorts. The GSE17538 (n=158) and GSE39582 (n=326) comprised CRC patients. Each dataset was subjected to every of the four machine learning classifiers based on the indicated gene signatures. For comparison between different immune populations, the classifier with the best performance (the highest AUC values) for most immune populations in a given dataset was defined as the focus model. Alternatively, to compare the performance of the EIM-based models with CellAge- or GenAge-based models, the optimal AUC value of each model was labeled. The EIM component subpopulations were marked in blue, and EIM was in red. EIM had a better performance than any individual component subpopulations.

**C**. Evaluation of model performance in classifying the elders using the age-related gene signatures cellage, geneage_up, and geneage_down from the commonly used CellAge database. These age-related gene signatures had a lower AUC than that of the EIM gene signature.

**
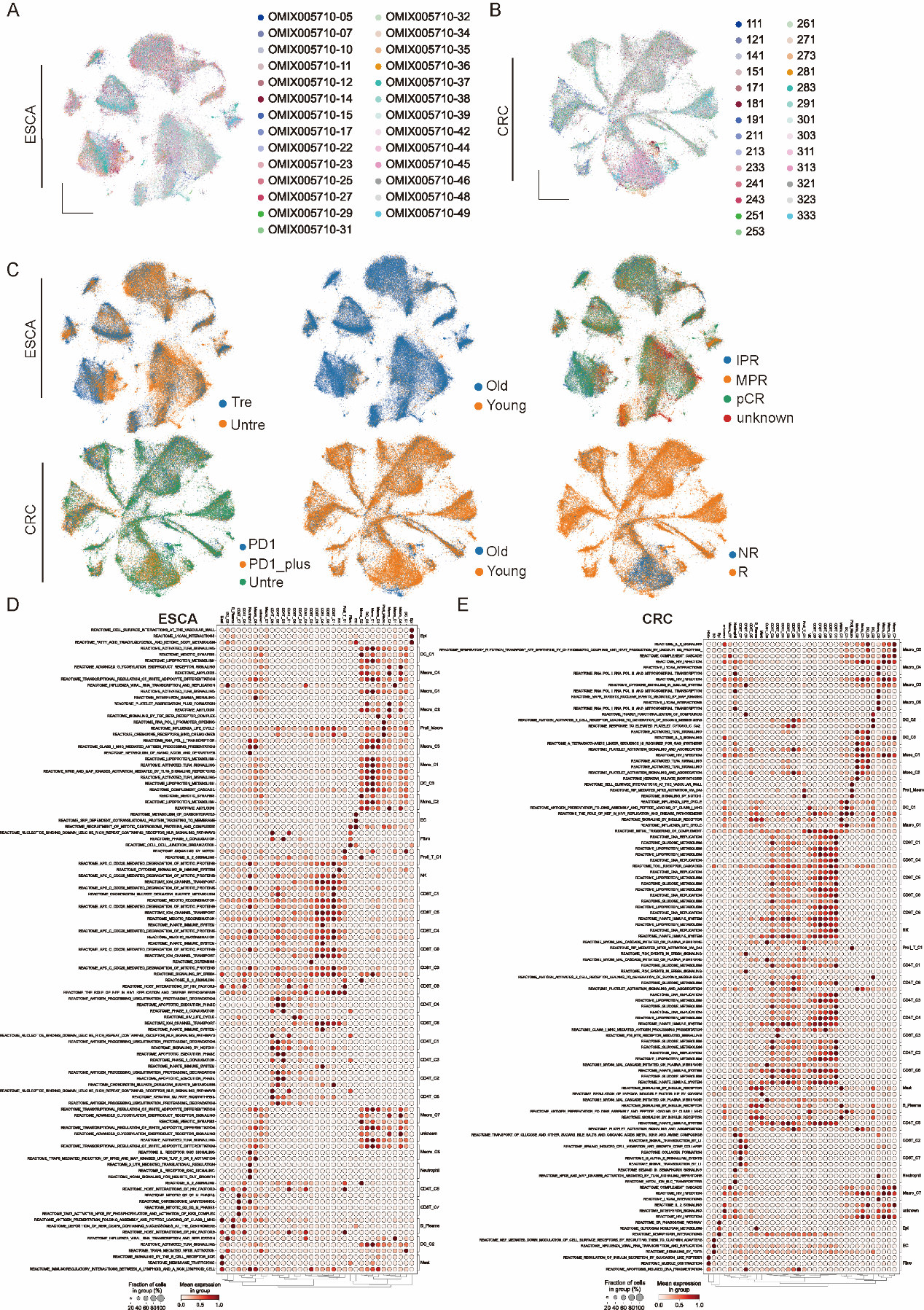
**

**Fig. S6. Construction of scRNA-seq atlas for ESCA and CRC patients treated with immunotherapy**

**A** and **B**. Minimum-Distortion Embedding (MDE) visualization of cell clusters in ESCA (**A**) and CRC (**B**) tumors. Public 10× Genomics scRNA-Seq GEO datasets for transcriptomes of ESCA (125,759 cells, n = 27 samples) and CRC (79,797 cells, n = 27 samples) were preprocessed and integrated with R package *Seurat* using classical pipelines, followed by removal of batch effect with the *Harmony* algorithm. Unsupervised clustering was performed based on classical cell markers.

1. Visualization of cell clusters in ESCA (upper) and CRC (lower) tumors showing the transcriptomes from patients with different treatments (left), of different age groups (middle), and with different responses (right).

**D** and **E**. Dot plot showing the top3 pathways enriched in the annotated clusters as analyzed by *AUCell*. Pathway scores were calculated using the scRNAseq data based on REACTOME databases. Dot size represents the fraction of annotated clusters with pathway expression. Dot color represents the scaled expression. The enriched pathway may indicate the functions of the annotated clusters.


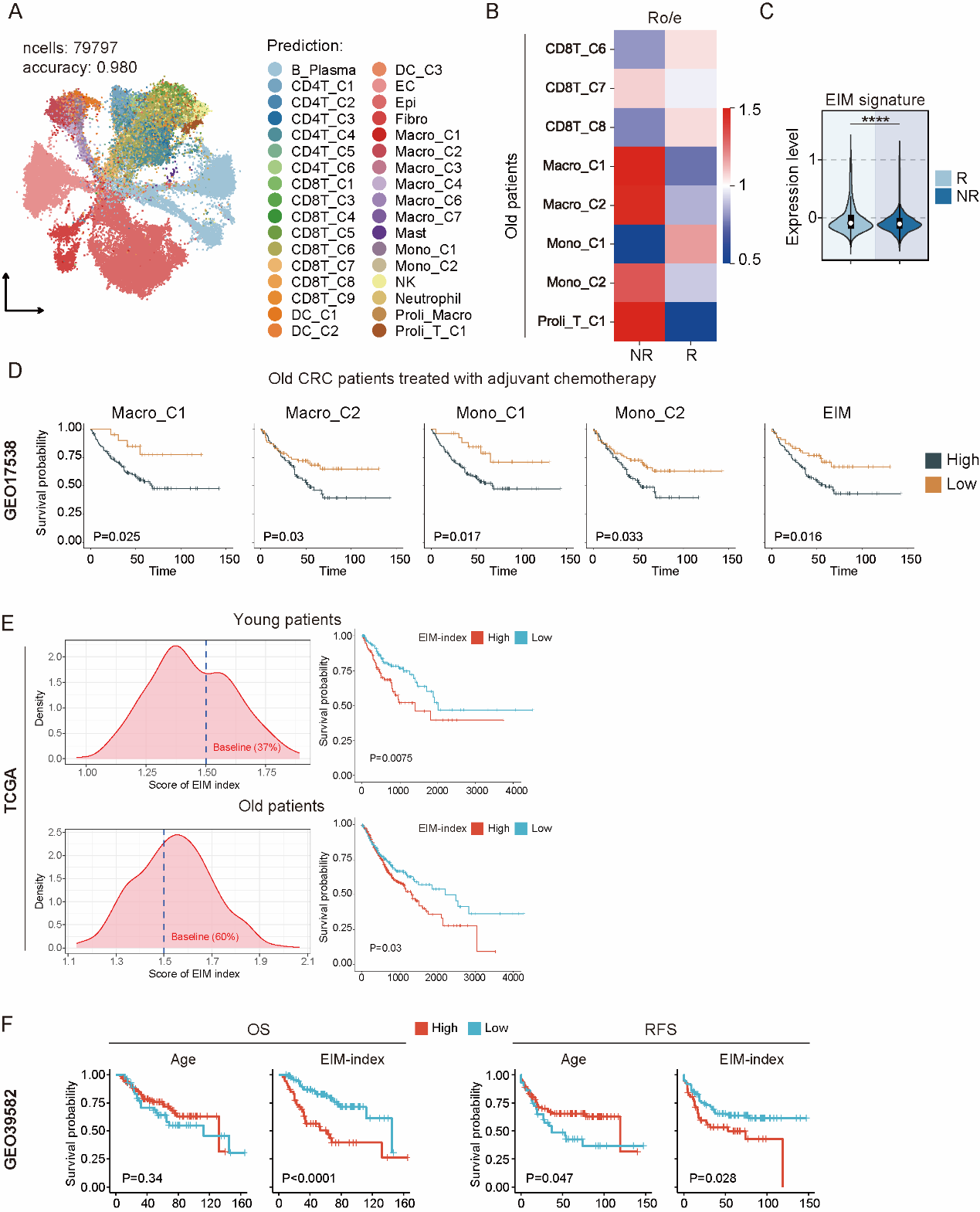


**Fig. S7. EIM inversely correlates to response to therapies and survivals**

**A**. Minimum-Distortion Embedding (MDE) visualization of cell clusters in tumors from CRC patients recruited for neoadjuvant immunotherapy with anti-PD1 mAb. Public 10× Genomics scRNA-Seq GEO datasets for transcriptomes of tumor tissues (79,797 cells, 27 samples) were processed, and cell clusters were identified as mentioned above. The mapping accuracy was 0.980.

**B**. Heatmap showing the Ro/e of EIM component subpopulations in older patients with different responses to treatment. The subpopulations with Ro/e > 1 were regarded as with preference for a specific group. Color represents Ro/e value. NR, no response; R, response. The color means Ro/e values.

**C**. Violin plot showing expression levels of the EIM gene signature in CRC patients with different responses to anti-PD1 mAb neoadjuvant therapy. Each patient was scored for the level of EIM gene signature with GSVA. P value is calculated with Wilcox test.

**D.** Correlation between the EIM or its component subpopulations and survivals of the older CRC patients treated with adjuvant chemotherapy in the GEO17538 dataset. The gene signatures for the EIM or its component subpopulations were used for calculation of a corresponding score for each patient with GSVA. Patients were further stratified into high and low groups followed by Kaplan-Meier analysis using R packages survival and survminer (log-rank test). The curves with a p value < 0.05 were shown.

**E.** Distribution of patients with different EIM index scores in the younger and older patient groups (left) and the matched survival plot (right) using the TCGA dataset of GI tract tumors. Density was calculated by ggpurb. The patients were scored for the EIM index by using GSVA. By setting a cut-off score of 1.5, 37% patients of the younger group and 60% patients of the older group were stratified to the high EIM index groups. Kaplan-Meier survival curve was generated subsequently using survival and survminer.

**F.** Kaplan-Meier survival analysis of CRC patients treated with adjuvant chemotherapy in the GEO39582 dataset. Each patient was scored for the EIM index using GSVA. Patients were automatically stratified into high and low groups and Kaplan-Meier curves were generated with the R package *survival*. RFS: relapse-free survival; OS: overall survival.
